# MRI-based radiomics nomogram for predicting CD8-positive tumor-infiltrating lymphocytes levels in HER2-positive breast cancer

**DOI:** 10.3389/fonc.2025.1612631

**Published:** 2025-10-10

**Authors:** Xiaoguang Li, Qiujie Dong, Chao Cong, Hong Guo, Chunlai Zhang, Peng Zhong, Jingqin Fang, Yi Wang

**Affiliations:** ^1^ Department of Radiology, Daping Hospital, Army Medical University, Chongqing, China; ^2^ Department of Nuclear Medicine, Daping Hospital, Army Medical University, Chongqing, China; ^3^ School of Electrical and Electronic Engineering, Chongqing University of Technology, Chongqing, China; ^4^ Department of Pathology, Daping Hospital, Army Medical University, Chongqing, China; ^5^ Department of Ultrasound, Daping Hospital, Army Medical University, Chongqing, China

**Keywords:** radiomics, magnetic resonance imaging, CD8-positive tumor-infiltrating lymphocytes, human epidermal growth factor receptor 2, breast cancer

## Abstract

**Objective:**

To develop a radiomics nomogram based on radiomic features derived from dynamic contrast-enhanced magnetic resonance imaging (DCE-MRI) combined with clinical-imaging characteristics in predicting the CD8+Tumor-infiltrating lymphocytes (TILs) levels in patients with human epidermal growth factor receptor 2 (HER2)-positive breast cancer (BC).

**Materials and methods:**

A total of 126 BC patients with pathologically confirmed HER2-positive were enrolled and randomly divided into training (n = 88) and validation (n = 38) cohorts. A clinical-imaging model was built based on clinical and MRI characteristics. Radiomics features were extracted from the third post-contrast phase on DCE-MRI. Select K Best, the maximum relevance minimum redundancy (mRMR), and least absolute shrinkage and selection operator algorithm (LASSO) were used to select radiomics features and a radiomics signature score (rad-score) was constructed by seven radiomics features. Multivariate logistic regression analysis was used to construct a radiomics nomogram model by combining with rad-score and independent clinical-imaging factors. Performance of the clinical-imaging model, rad-score, and radiomics nomogram model were evaluated using the area under the curve (AUC).

**Results:**

Seven radiomics features were used to build the rad-score. The rad-score achieved good performance in predicting CD8+TILs with AUCs= 0.853 and 0.822, respectively. The radiomics nomogram model based on rad-score and clinical-imaging features (tumor margin and enhancement pattern) yielded an optimal AUC of 0.866 and 0.886 in the training and validation cohorts, respectively. The radiomics nomogram significantly outperformed the clinical-imaging model (p < 0.05) and showed a trend toward better performance compared to the rad-score alone (p > 0.05).

**Conclusions:**

The MRI-based radiomics nomogram has the ability to predict CD8+TILs levels, which could be useful in identifying potential in HER2-positive BC patients who can benefit from immunotherapy.

## Introduction

Breast cancer (BC) is a highly heterogeneous malignant tumor with various biological characteristics associated with prognosis and treatment response. In recent years, immunotherapy and immune checkpoint blockade have gained significant attention in the clinical management of BC patients (1). In comparison with conventional treatment modalities such as chemotherapy and radiotherapy, immunotherapy has been shown to exhibit a reduced incidence of adverse effects and to be better tolerated by patients. However, the efficacy of immunotherapy varies, with only a subset of BC patients demonstrating a response. Therefore, predictive biomarkers are crucial for identifying BC patients who would truly benefit from immune-based therapies. Tumor-infiltrating lymphocytes (TILs) are essential components of the tumor immune microenvironment (TIME), which play an important role in tumor genesis, progression, metastasis, and drug resistance processes, and are considered as a biomarker of immune infiltration and prognosis of cancer patients. Current evidence suggests that the presence of TILs is associated with a favorable prognosis in BC and other solid tumors patients (2).

T lymphocytes, comprising CD8+ T cells, CD4+ helper T cells, and regulatory T cells, are the most widely distributed immune cell types within TILs. CD8+ T cells execute key cytotoxic functions within the TIME and mediate responses to immune checkpoint inhibitors (ICIs). In BC management, ICIs have shown value in improving patients’ clinical outcomes, and an increase in baseline density of CD8+ T cells within tumors is associated with a favorable response to immunotherapy (3). Among the different molecular subtypes of BC, human epidermal growth factor receptor 2 (HER2)-positive BC has been demonstrated to exhibit high immunogenicity, with approximately 55% containing high TILs levels in the stroma (4). A clinical trial has revealed that higher TILs levels in HER2-positive BC patients after neoadjuvant chemotherapy are strongly associated with increased rates of pathologic complete response and overall survival (OS) (5). Moreover, increased TILs have shown to be indicative of a better response to HER2-targeted therapy in HER2-positive BC patients (6). Hou et al. (7) has demonstrated a positive correlation between high CD8+TILs levels and prolonged OS in HER2-positive BC patients, suggesting that CD8+ T cells-mediated cytotoxic immune responses may predict better clinical outcomes after standard chemotherapy and HER2 blockade treatment. Consequently, precise assessment of CD8+TILs levels before surgery is important for guiding precise treatment strategies for patients with HER2-positive BC.

Currently, the assessment of CD8+TILs levels in BC relies on immunohistochemical (IHC) staining of tissue specimens obtained through surgical resection or needle biopsy. However, these invasive and non-reproducible measures carry the risk of trauma or complications, limiting their use in patients with poor clinical conditions. Therefore, finding an accurate, non-invasive and repeatable method to assess TILs levels may help predict clinical outcomes for HER2-positive BC patients who may benefit not only from neoadjuvant or anti-HER2-targeted therapies but also from immunotherapy.

Recently, radiomics, which involves the extraction and analysis of large numbers of quantitative image features from medical images, has emerged as a promising computational medical imaging technique (8). Dynamic contrast-enhanced magnetic resonance imaging (DCE-MRI) is the most sensitive imaging modality for detecting BC, making it suitable for clinical application in radiomics. Many studies have demonstrated the use of DCE-MRI radiomics in differentiating between benign and malignant breast tumors (9), molecular subtyping of BC, assessment of axillary lymph node status (10), and predicting prognosis and treatment response (11). However, to the best of our knowledge, there have been no studies using DCE-MRI radiomics to predict CD8+TILs levels in HER2-positive BC. The purpose of this study is to investigate the potential of a radiomics nomogram, constructed using a combination of clinical-imaging and DCE-MRI radiomics features, in predicting CD8+TILs levels in HER2-positive BC.

## Materials and methods

### Patients

**Figure 1:**
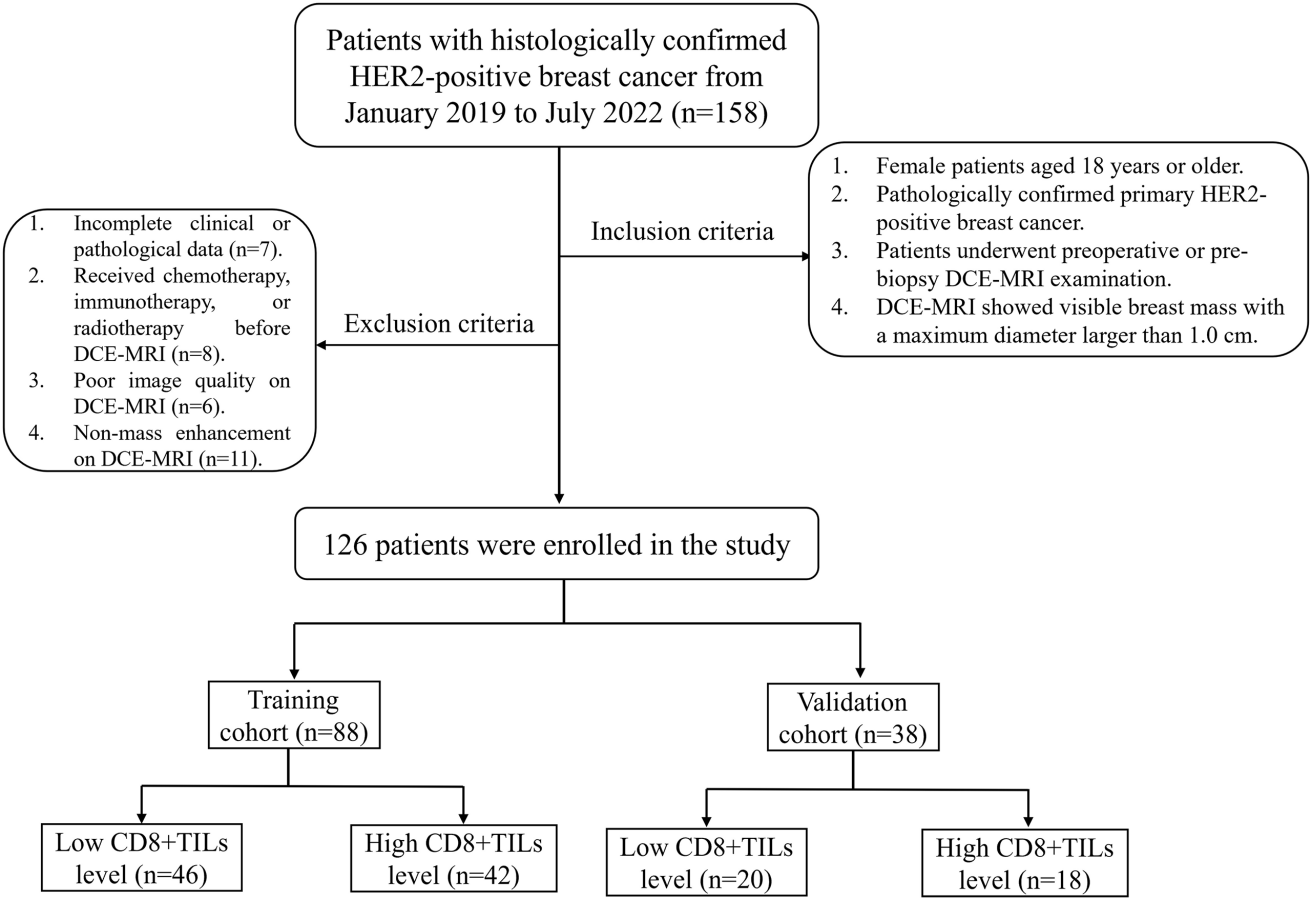
Flowchart of the study population.

This retrospective study was approved by the Ethics Committee of Daping Hospital [Approval No: 2023 (06)]. The clinical and imaging data of 158 BC patients with histologically confirmed HER2-positive from January 2019 to July 2022 were retrospectively collected, and 126 patients who met inclusion and exclusion criteria were enrolled in this study. The inclusion criteria included: (1) Female patients aged 18 years or older; (2) Pathologically confirmed primary HER2-positive breast cancer; (3) Patients underwent preoperative or pre-biopsy DCE-MRI examination; (4) DCE-MRI showed visible breast mass with a maximum diameter larger than 1.0 cm. The exclusion criteria included: (1) Incomplete clinical or pathological data; (2) Received chemotherapy, immunotherapy, or radiotherapy before DCE-MRI; (3) Poor image quality on DCE-MRI; (4) Non-mass enhancement on DCE-MRI. Patients were randomly divided into a training cohort (n=88) and a validation cohort (n = 38) in a ratio of 7:3. The flow chart was shown in **Figure 1**.

### MRI technique

All patients underwent bilateral breast MRI using a 1.5T scanner (Magnetom Aera, Siemens Healthcare, Erlangen, Germany). The MRI protocol included: T1WI (TR/TE=8.6ms/4.7ms, FOV = 360mm×360mm, matrix size=384×384, Slice thickness=4.0mm); Fat saturation T2WI (TR/TE=5600ms/57ms, FOV = 340mm×340mm, matrix size=320×320, Slice thickness=4.0mm); DCE-T1WI (TR/TE=4.62ms/1.75ms, FOV = 360mm×360mm, matrix size=320×320, Slice thickness=1.5mm). Gd-DTPA (Magnevist, Bayer Healthcare, Berlin, Germany) was used as a contrast agent during the enhanced scan and was injected into the elbow vein by a high-pressure syringe at a dose of 0.1 mmol/kg and a flow rate of 2.0 ml/s. Then, 15ml of normal saline was injected at the same flow rate after the contrast agent injection. A total of seven phases (one basal pre-contrast and six continuous post-contrast phases) were continuously collected without intervals. Each scanning duration was approximately 60 seconds, a total of about 7 minutes.

### Clinical data

The following clinical data were collected: age, menopausal status, clinical TNM stage, histologic type, histologic grade, Ki67 expression (low expression<20%; high expression≥20% (12)), estrogen receptor (ER) status, and progesterone receptor (PR) status. ER and PR positive were defined as nuclear staining of at least 1% of tumor cells (13).

The evaluation of CD8+TILs followed the recommendations of the International TILs Working Group in 2014 (14). The whole slide was scanned at low magnification (50×), and CD8+TILs were identified by a faint yellow to brown coarse granule staining on the cell membrane. Then, five high-power fields (200×) were randomly selected to evaluate CD8+TILs expression based on the percentage of positive lymphocytes in the tumor stroma, and the average value was calculated as the average level of CD8+TILs in the entire tumor area. Hotspot regions were avoided during the analysis process. As there is currently no consensus on the threshold for CD8+TILs, the median count was used as the cutoff value (15). The median of CD8+TILs in this study was 30%, so CD8+TILs expression ≤ 30% was low level and marked as (-), and > 30% was high level and marked as (+). All IHC slides were jointly analyzed by two pathologists with 8 and 10 years of experience in breast pathology at our hospital, and disagreements were resolved through consultation. Both pathologists were unaware of the clinical and imaging data before reviewing the slides.

### MRI interpretation

Blinded to the clinical and histopathological findings, two radiologists (reader 1 and 2, with 10 and 8 years of breast imaging experience, respectively) independently reviewed the MRI data. When compiling the statistics, if it was found that the opinions of the two readers were inconsistent, then a consensus would be reached through negotiation and discussion. MRI features were recorded based on BI-RADS MRI lexicon (16–18), including tumor maximum diameter (which was measured at the largest slice of the tumor), shape (oval, round and irregular, with lobulated consolidated into oval), margin (smooth margins were circumscribed, irregular and spiculated margins were not circumscribed), background parenchymal enhancement (BPE) [visual estimation of normal background parenchymal enhancement seen with the first contrast-enhanced sequence was classified as minimal (<25%), mild (25%-50%), moderate (51%-75%) or marked (>75%), enhancement pattern (homogenous was confluent uniform enhancement within the entire mass; heterogeneous was non-uniform with variable signal intensity; rim enhancement was more pronounced enhancement towards the periphery than the center.), time-intensity curve (TIC) pattern (persistent was defined as a continued increase in signal intensity of more than 10% over time; plateau remained a qualitative description, defined as signal intensity that does not change over time after the initial rise; washout was defined as a decrease of more than 10% from the highest signal intensity during the initial rise), and axillary lymph node size [selected the largest axillary lymph node in the fat saturation T2WI sequence, measured its maximal cross-sectional area by manually delineating the entire boundary of the lymph node on the largest slice using the picture archiving and communication system (PACS)].

### Tumor segmentation and feature extraction

As previous study (19)suggested, the contrast between breast malignant lesions and the surrounding glandular background occurs its peck about 60–180 seconds after injection of contrast agent. In this study, the third post-contrast phase on DCE-MRI (DCE-MRI_phase3_) was selected for tumor segmentation. First, the “N4 Bias Field Correction” and “Image Intensity Filter” module plugged in 3D-Slicer software (Version 4.11.20210226, https://www.slicer.org/) were used to correct the bias filed and normalize the image intensity. Radiologist 1 manually delineated each layer of regions of interest (ROI) by contouring the tumor along its boundaries to obtain three-dimensional volumes of interest (VOI) by 3D-Slicer software. Radiologist 2 delineated the ROIs for 40 randomly selected tumors. Radiologist 1 repeated the same procedure for a second ROI depiction from the randomly selected images after 1 month. During the delineation process, efforts were made to exclude normal tissues surrounding the tumor but include areas of hemorrhage, and necrosis. All VOIs were resampled to a voxel size of 1.0 × 1.0 × 1.0 mm, and the bin width of the grayscale histogram was fixed at 25 for image discretization. DCE-MRI images were processed using five Laplacian Gaussian filters (i.e., the kernel size μ was set to 1, 2, 3, 4, 5) and filters based on wavelet variations (LLL, LLH, LHL, HLL, LHH, HLH, HHL, HHH). Radiomics feature extraction was performed using the open-source software package “Pyradiomics” on Python 3.7 (https://www.python.org/), which included seven major categories: shape feature, histogram, gray-level co-occurrence matrix (GLCM), gray-level run length matrix (GLRM), gray-level size zone matrix (GLSZM), neighboring gray tone difference matrix (NGTDM), and gray-level dependence matrix (GLDM). A total of 1218 radiomics features were extracted from each VOI. These features conformed to the guidelines of the Image Biomarker Standardization Initiative Reference Manual (ISBI).

### Radiomics nomogram construction

Only radiomics features with intra-observer intraclass correlation coefficient (ICC) and inter-observer ICC greater than 0.75 were used for further analysis. Using the Darwin research platform (https://www.yizhun-ai.com/), four-step procedure was used to select the final radiomic features of the training cohort: (a) all features were preprocessed by maximum absolute normalization; (b) Use the “Select K Best” feature selection module to reduce the feature dimension; (c) The maximum relevance minimum redundancy (mRMR) algorithm was used to retain top 20 features; (d) Finally, the least absolute shrinkage and selection operator (LASSO) with 5-fold cross-validation was used to filter out the optimal features.

In the training cohort, the selected radiomics features and their corresponding LASSO regression coefficients were combined in a weighted linear to establish the Rad-score, and the Rad-score of each tumor was analyzed by univariate logistic regression to construct the radiomics model. Meanwhile, univariate and multivariate logistic regression analyses were performed to identify independent clinical-imaging factors. Only factors with P<0.05 in univariate analysis were incorporated into multivariate analysis. These prominent factors were used to construct a clinical-imaging model. Finally, a radiomics nomogram model was constructed using multivariate logistic regression combined with clinical-imaging features and Rad-score. The performance of each model built by the training cohort was verified in the verification cohort.

### Statistical analysis

Statistical analysis was performed using SPSS and R software. The normality and homogeneity of variance of the data were evaluated using the Kolmogorov-Smirnov test and Levene’s test. Normally distributed continuous data were presented as mean ± standard deviation (SD), while non-normally distributed data were presented as median (interquartile range). Independent sample t-tests or Mann-Whitney U tests were used for intergroup comparisons. Categorical data were presented as frequencies and percentages, and intergroup comparisons were performed using the chi-square test or Fisher’s exact test. The receiver operating characteristic (ROC) curve was plotted using Medcalc 20.0, and the area under the curve (AUC), sensitivity, specificity, and accuracy were calculated to evaluate model’s performance. Delong test was used to compare the AUC differences among different models. ICCs were calculated using the “psych” package, calibration plots were performed using the “rms” package, and decision curve analysis (DCA) was performed using the “devtools” package. p<0.05 was considered statistically significant.

## Results

### Patient characteristics and clinical-imaging model

**Figure 2:**
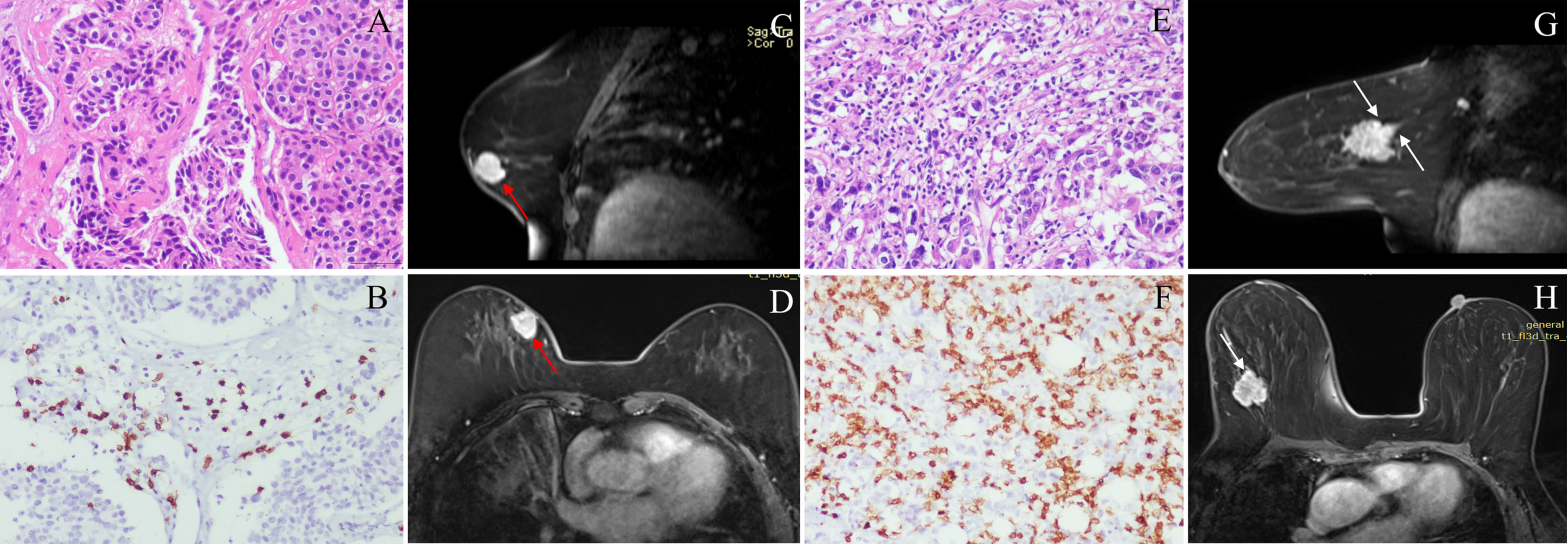
Classic MRI features of breast cancers with low and high CD8+TILs levels. A HER2-positive breast cancer (**(A)** HE ×200) with low CD8+TILs level (about 20%) (**(B)** IHC×200) presented an oval mass with circumscribed margin (red arrow) and homogeneous enhancement on DCE-MRI **(C, D)**. A HER2-positive breast cancer (**(E)** HE ×200) with high CD8+TILs level (about 80%) (**(F)** IHC×200) presented an oval mass with not circumscribed margin (white arrow) and heterogeneous enhancement on DCE-MRI **(G, H)**. MRI, magnetic resonance imaging; TILs, Tumor-infiltrating lymphocytes; HER2, human epidermal growth factor receptor 2; HE, hematoxylin and eosin staining; IHC, immunohistochemical; DCE-MRI: dynamic contrast-enhanced magnetic resonance imaging.

**Figure 3:**
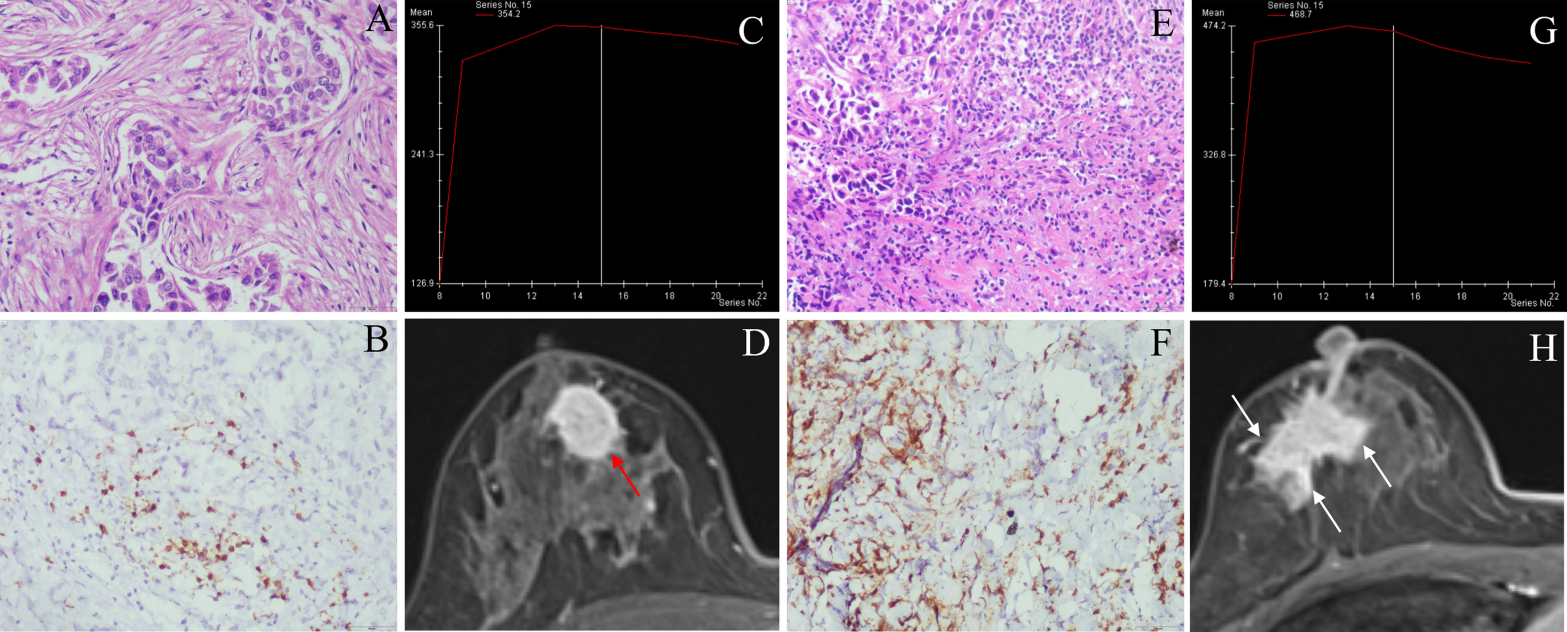
Classic MRI features of breast cancers with low and high CD8+TILs levels. A HER2-positive breast cancer (**(A)** HE ×200) with low CD8+TILs level (about 10%) (**(B)** IHC×200) presented an oval mass with circumscribed margin (red arrow), and the TIC curve was plateau on DCE-MRI **(C, D)**. A HER2-positive breast cancer (**(E)** HE ×200) with high CD8+TILs level (about 60%) (**(F)** IHC×200) presented an irregular mass with not circumscribed margin (white arrow), and TIC curve was washout on DCE-MRI **(G, H)**. MRI, magnetic resonance imaging; TILs, Tumor-infiltrating lymphocytes; HER2, human epidermal growth factor receptor 2; HE, hematoxylin and eosin staining; IHC, immunohistochemical; DCE-MRI: dynamic contrast-enhanced magnetic resonance imaging, TIC, time-intensity curve.

52.4% of patients (66/126) were low CD8+TILs level and 47.6% (60/126) were high CD8+TILs level. BC patients’ clinical and imaging features in the training and validation cohorts were shown in **Tables 1** and **2**. Among these features, there were significant differences in N stage, maximum diameter, tumor margin, and enhancement pattern in the training and validation cohorts, and T stage in the training cohort (p < 0.05). Classic MRI features of HER2-positive BC with low and high CD8+TILs levels were shown in **Figure 2** and **Figure 3**. In the training cohort, univariate analysis showed significant associations between several risk factors and CD8+TILs levels, including T stage, N stage, maximum diameter, tumor margin, and enhancement pattern (p < 0.05). Multivariate regression analysis revealed that tumor margin and enhancement pattern were independent risk factors (p < 0.05). The AUC of the clinical-imaging model constructed by these two variables was 0.785 [95% confidence interval (CI): 0.690-0.881] and 0.803 (95% CI: 0.654-0.951) in the training and validation cohorts, respectively.

**Table 1:** Comparison of clinical features between HER2-positive breast cancer patients with low and high CD8+TILs levels in the training and validation cohorts.

Variables	Training cohort (n=88)		P value	Validation cohort (n=38)		P value
	Low CD8+TILs level (n=46)	High CD8+TILs level (n=42)		Low CD8+TILs level (n=20)	High CD8+TILs level (n=18)	
Age (year), mean ± SD	49.57 ± 11.06	54.19 ± 10.72	0.051	51.85 ± 6.91	52.61 ± 9.91	0.783
Menopause			0.157			0.757
No	21(45.7%)	13(31.0%)		10(50.0%)	10(55.6%)	
Yes	25(54.3%)	29(69.0%)		10(50.0%)	8(44.4%)	
T stage			0.002			0.051
Tis	2(4.3%)	0(0.0%)		2(10.0%)	0(0.0%)	
T1	3(6.5%)	1(2.4%)		2(10.0%)	1(5.6%)	
T2	37(80.4%)	27(64.3%)		15(75.0%)	11(61.1%)	
T3	2(4.3%)	6(14.3%)		1(5.0%)	5(27.8%)	
T4	2(4.3%)	8(19.0%)		0(0.0%)	1(5.6%)	
N stage			0.028			0.009
N0	23(50.0%)	12(28.6%)		10(50.0%)	2(11.1%)	
N1	17(37.0%)	19(45.2%)		9(45.0%)	11(61.1%)	
N2	3(6.5%)	5(11.9%)		1(5.0%)	3(16.7%)	
N3	3(6.5%)	6(14.3%)		0(0.0%)	2(11.1%)	
Metastasis			0.315			0.395
M0	38(82.6%)	33(78.6%)		18(90.0%)	14(77.8%)	
M1	1(2.2%)	4(9.5%)		2(10.0%)	4(22.2%)	
Mx	7(15.2%)	5(11.9%)		0(0.0%)	0(0.0%)	
Histologic type			0.465			0.488
Non-invasive	4(8.7%)	2(4.8%)		2(10.0%)	0(0.0%)	
Invasive	42(91.3%)	40(95.2%)		18(90.0%)	18(100.0%)	
Histologic grade			0.368			0.633
I	0(0.0%)	0(0.0%)		0(0.0%)	0(0.0%)	
II	36(78.3%)	36(85.7%)		17(85.0%)	17(94.4%)	
III	10(21.7%)	6(14.3%)		3(15.0%)	1(5.6%)	
Ki-67			0.974			0.606
Low	13(28.3%)	12(28.6%)		3(15.0%)	1(5.6%)	
High	33(71.7%)	30(71.4%)		17(85.0%)	17(94.4%)	
ER status			0.177			1.000
Negative	23(50.0%)	2764.3%)		12(60.0%)	11(61.1%)	
Positive	23(50.0%)	15(35.7%)		8(40.0%)	7(38.9%)	
PR status			0.572			0.522
Negative	28(60.9%)	28(66.7%)		11(55.0%)	12(66.7%)	
Positive	18(39.1%)	14(33.3%)		9(45.0%)	6(33.3%)	

HER2, human epidermal growth factor receptor 2;TILs, Tumor-infiltrating lymphocytes; SD, standard deviation; ER, estrogen receptor; PR, progesterone receptor.

**Table 2:** Comparison of imaging features between HER2-positive breast cancer patients with low and high CD8+TILs level s in the training and validation cohorts.

Variables	Training cohort (n=88)		P value	Validation cohort (n=38)		P value
	Low CD8+TILs level (n=46)	High CD8+TILs level (n=42)		Low CD8+TILs level (n=20)	High CD8+TILs level (n=18)	
Maximum diameter(cm), mean ± SD	2.56 ± 0.91	3.05 ± 1.10	0.023	2.38(1.93,2.69)	3.44(2.45,4.56)	0.002
Shape			0.572			1.000
Round/oval	44(95.7%)	39(92.9%)		19(95.0%)	17(94.4%)	
Irregular	2(4.3%)	3(7.1%)		1(5.0%)	1(5.6%)	
Margin			0.001			0.028
Circumscribed	36(78.3%)	19(45.2%)		13(65.0%)	5(27.8%)	
Not circumscribed	10(21.7%)	23(54.8%)		7(35.0%)	13(72.2%)	
Enhancement pattern			0.000			0.022
Rim	7(15.2%)	8(19.0%)		1(5.0%)	7(38.9%)	
Heterogeneous	12(26.1%)	28(66.7%)		10(50.0%)	8(44.4%)	
Homogeneous	27(58.7%)	6(14.3%)		9(45.0%)	3(16.7%)	
BPE			0.787			1.000
Minimal (<25%)	28(60.9%)	26(61.9%)		7(35.0%)	5(27.8%)	
Mild (25%-50%)	12(26.1%)	12(28.6%)		9(45.0%)	11(61.1%)	
Moderate (51%-75%)	3(6.5%)	4(9.5%)		4(20.0%)	2(11.1%)	
Marked (>75%)	3(6.5%)	0(0.0%)		0(0.0%)	0(0.0%)	
TIC pattern			0.387			0.103
I (persistent)	2(4.3%)	0(0.0%)		0(0.0%)	0(0.0%)	
II (plateau)	27(58.7%)	25(59.5%)		7(35.0%)	12(66.7%)	
III (washout)	17(37.0%)	17(40.5%)		13(65.0%)	6(33.3%)	
ALN size (cm^2^), mean ± SD	2.22 ± 2.34	3.07 ± 3.29	0.166	4.06 ± 9.88	4.59 ± 3.60	0.832

HER2, human epidermal growth factor receptor 2;TILs, Tumor-infiltrating lymphocytes; SD, standard deviation; BPE, background parenchymal enhancement; TIC, time-intensity curve; ALN, axillary lymph node.

### Features selection and rad-score model

**Figure 4:**
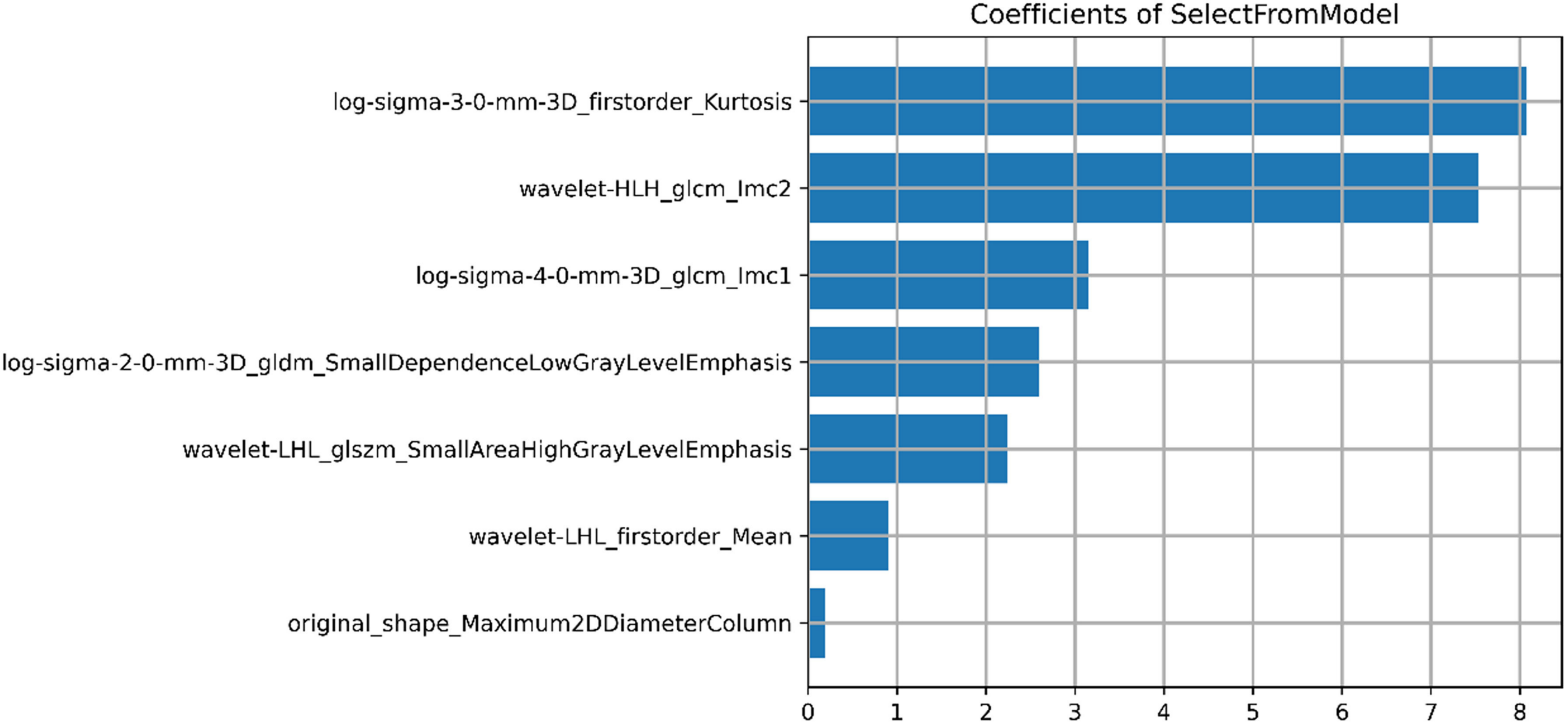
Result of selected radiomics features. Seven radiomics features were presented, including 1 morphological feature, 2 first-order statistical features, and 4 texture features.

**Figure 5:**
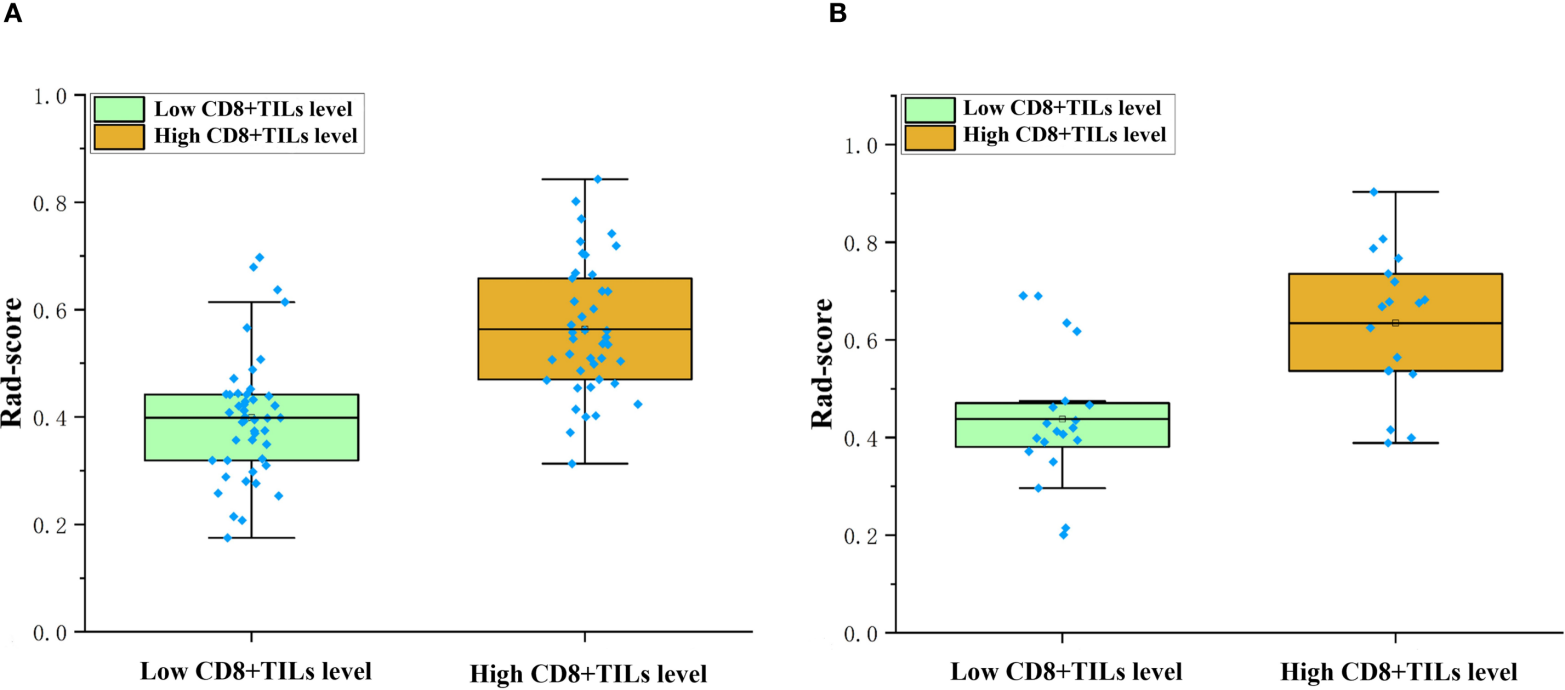
Comparison of rad-score between different CD8+TILs levels. High CD8+TILs level had higher rad-score than the low CD8+TILs level in the training **(A)** and validation cohorts **(B)** (p < 0.001). TILs, Tumor-infiltrating lymphocytes.

A total of 1218 radiomics features were extracted from each VOI. After removing features with inter-observer and intra-observer ICCs ≤ 0.75, 1093 features were obtained. In the training cohort, the Select K Best algorithm and mRMR algorithm were used for dimensionality reduction. Finally, 7 features were selected by the LASSO regression algorithm, including 1 shape feature, 2 first-order statistical features, and 4 texture features (**Figure 4**). In both the training and validation cohorts, the Rad-score in high CD8+TILs level was significantly higher than the low CD8+TILs level (p < 0.001) (**Figure 5**). The Rad-score model demonstrated good performance in predicting CD8+TILs levels, with AUC of 0.853 (95% CI: 0.771-0.935) and 0.822 (95% CI: 0.686-0.958) in the training and validation cohorts, respectively. The Rad-score formula was as follows:

Rad-score=1.326×log-sigma-3-0-mm-3D_firstorder_Kurtosis-1.236×log-sigma-2-0-mm-3D_gldm_SmallDependenceLowGrayLevelEmphasis+1.075×wavelet-LHL_glszm_SmallAreaHighGrayLevelEmphasis+1.039×wavelet-LHL_firstorder_Mean +0.960×wavelet-HLH_glcm_Imc2 + 0.833×original_shape_Maximum2DDiameterColumn-0.760×log-sigma-4-0-mm-3D_glcm_Imc1-1.770.

### Radiomics nomogram model

**Figure 6:**
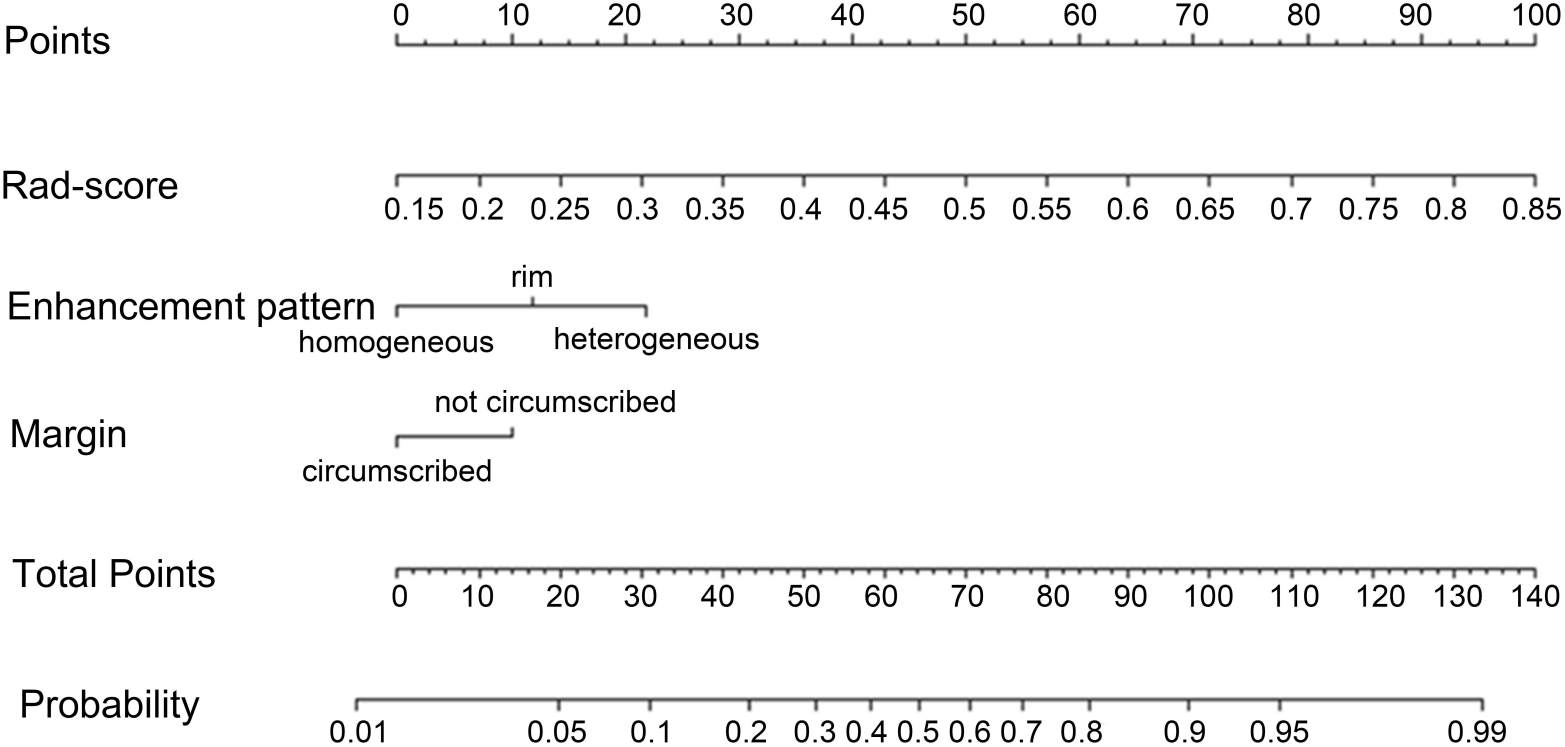
Radiomics nomogram. Radiomics nomogram constructed by rad-score and clinical-imaging features (tumor margin and enhancement pattern) for predicting high CD8+TILs level in the training cohort. TILs, Tumor-infiltrating lymphocytes.

**Figure 7:**
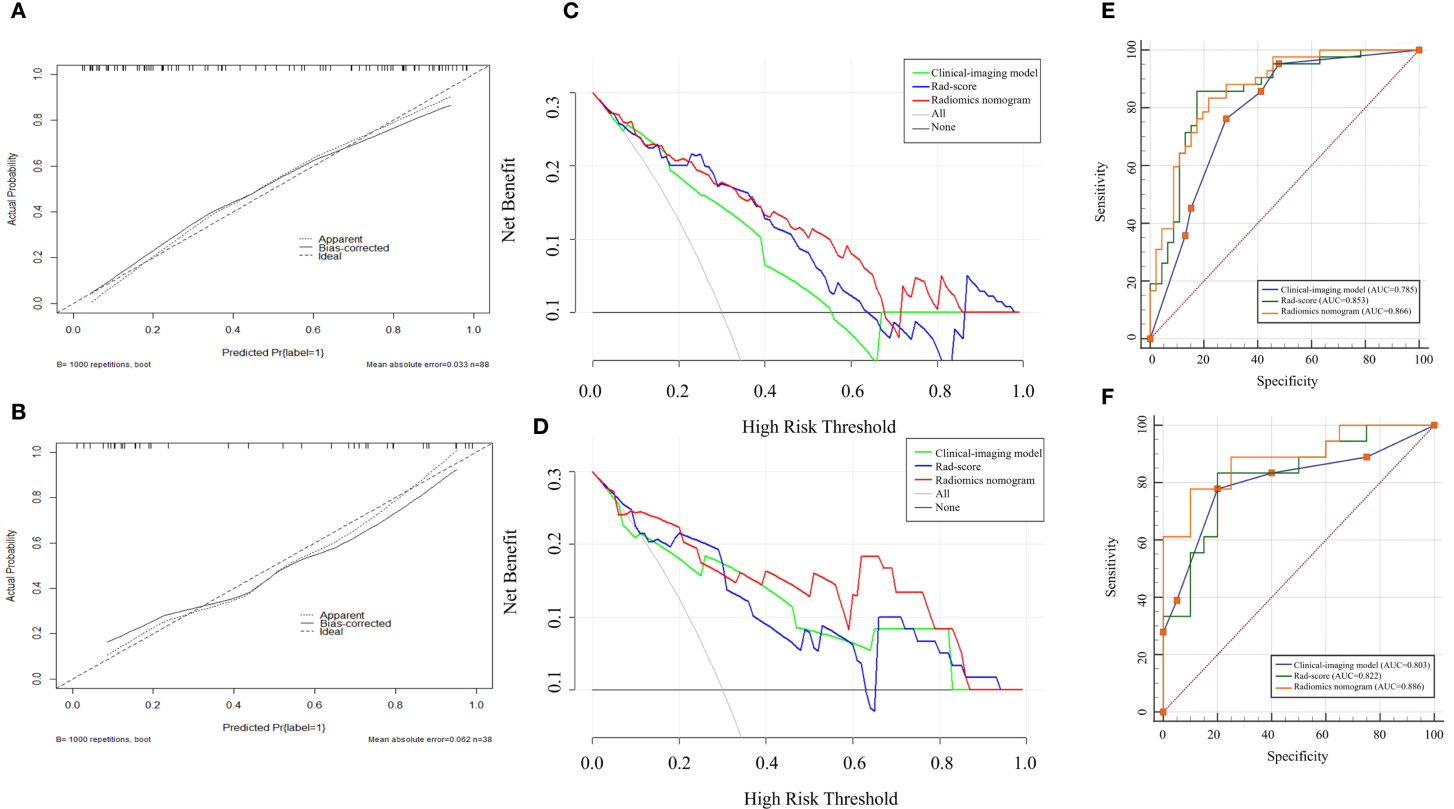
Calibration curves, DCA and ROC curves for different models. Calibration curves in the training **(A)** and validation **(B)** cohorts demonstrated the excellent discriminatory ability of the radiomics nomogram (the black solid line fitted to the diagonal ideal line). DCA in the training **(C)** and validation **(D)** cohorts revealed that the radiomics nomogram showed good clinical practicability and achieved the greatest net benefit. ROC curves in the training **(E)** and validation **(F)** cohorts showed radiomics nomogram had the highest AUC. DCA, decisive curve analysis, ROC, receiver operating characteristic; AUC, area under the curve.

The radiomics nomogram model was constructed based on the selected clinical-imaging risk factors (tumor margin and enhancement pattern) and rad-score (**Figure 6**). Calibration curves (**Figure 7**) showed the probability values of high CD8+TILs levels predicted by the radiomics nomogram model was in good agreement with the true values. Hosmer-Lemeshow test showed that the radiomics nomogram model was well calibrated in the training (p = 0.837) and validation (p = 0.600) cohorts. DCA showed that the radiomics nomogram model was more valuable in clinical application than clinical-imaging model and rad-score model (**Figure 7**). **Table 3** summarized the effectiveness of different models in predicting CD8+TILs levels. ROC curves of the three models for both the training and validation cohorts were shown in (**Figure 7**). The radiomics nomogram model achieved optimal predictive performance, with AUC of 0.866 (95% CI: 0.792-0.941) and 0.886 (95% CI: 0.778-0.994) in the training and validation cohorts, respectively. In the training cohort, Delong test showed that the radiomics nomogram model had a significantly higher diagnostic performance than the clinical-imaging model (p = 0.016), whereas there was no significant difference between the radiomics nomogram and Rad-score models (p = 0.550) (**Table 4**).

**Table 3:** Different models predict the effectiveness of CD8+TILs levels in HER2-positive breast cancer.

Models	Training cohort (n=88)			Validation cohort (n=38)		
	AUC (95% CI)	Sensitivity (%)	Specificity (%)	AUC (95% CI)	Sensitivity (%)	Specificity (%)
Clinical-imaging model	0.785 (0.690,0.881)	76.2%	71.7%	0.803 (0.654, 0.951)	77.8%	80.0%
Rad-score	0.853 (0.771,0.935)	85.7%	82.6%	0.822 (0.686, 0.958)	83.3%	80.0%
Radiomics Nomogram model	0.866 (0.792,0.941)	83.3%	78.3%	0.886 (0.778, 0.994)	77.8%	90.0%

TILs, Tumor-infiltrating lymphocytes; HER2, human epidermal growth factor receptor 2; AUC, area under the curve; CI, confidence interval.

**Table 4:** Comparison of AUC values in different models.

Models	Training cohort (n=88)		Validation cohort (n=38)	
	Z value	P value	Z value	P value
Clinical-imaging model vs Rad-score	1.263	0.206	0.222	0.825
Clinical-imaging model vs Radiomics nomogram model	2.412	0.016	1.398	0.162
Rad-score vs Radiomics nomogram model	0.598	0.550	1.376	0.169

AUC, area under the curve.

## Discussion

In this study, we established and validated an MRI-based radiomics nomogram by incorporating Rad-score and conventional MRI features to predict the CD8+TILs levels in HER2-positive BC. The radiomics nomogram model exhibited favorable performance for differentiating low CD8+TILs from high CD8+TILs levels in the HER2-positive BC, with an AUC of 0.866 and 0.886 in the training and validation cohorts, respectively. These findings suggest that the MRI-based radiomics nomogram could serve as an economical, effective and non-invasive tool for stratifying HER2-positive BC patients who may benefit from immunotherapy.

In this study, tumors exhibiting elevated levels of CD8+TILs demonstrated a significantly higher clinical T-stage and N-stage, suggesting that these tumors possessed a higher degree of malignancy. In terms of morphology, this study found that tumors with higher levels of CD8+ TILs tended to exhibit longer diameters, more not circumscribed margin, and heterogeneous enhancement. However, it should be noted that there were some discrepancies between the conclusions of other researchers and those of this study. Pujani et al. (20) believed that the TILs level of breast cancer larger than 5 cm was significantly elevated, and the proportion of HER2-positive breast cancer subtypes was 16.83% (17/101) in their study population. Bian et al. (21) concluded that the smaller tumor diameter was associated with higher TILs level, and the proportion of HER2-positive breast cancer was only 7.14% (11/154) in their study population. The reason why our research conclusion was similar to the former but different from the latter might be that the study populations we included were all patients with HER2-positive breast cancer. Choi et al. (22) observed that there was no association between tumor margin and TILs level, but they divided the TIL levels into three groups (low: <10%, intermediate: 10-50%, high: >50%) among patients with ER-negative/HER2-positive BC. Furthermore, Celebi et al. (23) observed that tumors exhibiting high TILs demonstrated more homogeneous enhancement compared to those with low TILs. However, in Celebi’s study, the proportion of HER2-positive breast cancer was only 5.06% (8/158), and enhancement pattern was not identified through the multivariate logistic regression analysis. What was most notable was that their study included different type breast cancers and only focused on TILs, not CD8+TILs. Utilizing univariate and multivariate logistic regression analyses, we identified tumor margin and enhancement pattern as independent predictors of high CD8+TILs levels. The clinical-imaging model, constructed on these two factors, demonstrated an area under the curve (AUC) of 0.785 (95% confidence interval [CI]: 0.690-0.881) in the training cohort and 0.803 (95% CI: 0.654-0.951) in the validation cohort.

Radiomics has recently emerged as a prevalent research tool in the field of tumor studies. This methodology involves the extraction of high-throughput quantitative features from medical images, facilitating the identification of subtle changes within tumors that are challenging to discern through visual assessment. A few previous studies have used radiomics to predict the TILs level in BC. Yu et al. (24) developed a radiomics model from digital mammograms, demonstrating excellent predictive performance for TILs level in both the training (AUC = 0.830) and validation cohorts (AUC = 0.790). Tang et al. (25) reported that features extracted from the delayed phase MRI, particularly DCE_Phase6_, provided superior information regarding the extent of TILs infiltration in comparison to features from other phases. However, these previous works only discussed overall TILs levels within all molecular subtypes of BC. Conversely, this study is the first attempt to develop a radiomics nomogram based on DCE-MRI _Phase3_ images for predicting the CD8+TILs levels in HER2-positive BC.

In this study, the rad-score model was constructed using seven features, which were primarily derived from Gaussian filtering and wavelet transformation of the original images. In both the training and validation cohorts, the rad-score of the high CD8+TILs level group was significantly higher than that those of the low CD8+TILs level group. These findings could be attributed to the fact that CD8+TILs are cytotoxic T cells that produce gamma interferon, which generates a tumor inflammatory environment such as central necrosis and edema (26). Thus, tumors with higher levels of CD8+TILs tend to have a more heterogeneous enhancement pattern. In this study, the rad-score model achieved an AUC of 0.853 in the training cohort and 0.822 in the validation cohort to stratify high and low levels of CD8+TILs. Arefan et al. (27) used imaging and gene expression data from 73 BC patients in the TCIA and TCGA databases to establish the relationship between radiomics features and tumor immune cell abundance by constructing a multivariate logistic regression model. The results showed that the predicted AUC of CD8+ T cell abundance was 0.740 and 0.620 in the cross-validation and external independent validation cohorts, respectively. The higher AUC for predicting CD8+TILs levels in this study may be attributed to the fact that the study focused on a particular BC molecular subtype and included more patient samples.

This study showed that the radiomics nomogram model based on rad-score and clinical-imaging features (tumor margin and enhancement pattern) exhibits optimal performance in predicting the CD8+TILs levels in HER2-positive BC. In the training cohort, the efficiency of the radiomics nomogram model was significantly higher than clinical-imaging model, and the difference was statistically significant (p = 0.016). Although the radiomics nomogram model was slightly higher than the rad-score, there was no statistical significance between them (p > 0.05). This finding indicated that radiomics features were strong components of the radiomics nomogram, while conventional imaging data were of limited value for model improvement.

There are several limitations in this study. Firstly, the study is retrospective, and there may be inherent biases in data collection. Secondly, this study is a single-center study with a limited number of cases and lacks external validation. Thirdly, this study lacks information on patient treatment response and prognosis. Future studies will further explore the relationship between DCE-MRI radiomic features and treatment response or prognosis in HER2-positive breast cancer patients with different CD8+TILs levels in the multi-center to enhance the clinical applicability of our findings.

## Conclusion

In conclusion, this study shows that the MRI-based radiomics nomogram has a good performance for predicting the CD8+TILs levels in HER2-positive BC. Our findings indicate a promising future for the guiding of immunotherapy in HER2-positive BC patients and deserve further in-depth study.

## Data Availability

The raw data supporting the conclusions of this article will be made available by the authors, without undue reservation.
